# Revealing Majorana Zero Modes in Vortex Cores via Nonmagnetic Impurities

**DOI:** 10.34133/research.1087

**Published:** 2026-01-23

**Authors:** Vyacheslav D. Neverov, Tairzhan Karabassov, Andrey V. Krasavin, Dimitri Roditchev, Vasily S. Stolyarov, Alexei Vagov

**Affiliations:** ^1^ Moscow Institute of Physics and Technology, Dolgoprudny 141700, Russian Federation.; ^2^ HSE University, Moscow 101000, Russian Federation.; ^3^ National Research Nuclear University MEPhI, Moscow 115409, Russian Federation.; ^4^ LPEM, UMR-8213, ESPCI Paris, PSL, CNRS, Sorbonne University, 75005 Paris, France.

## Abstract

Majorana zero modes (MZMs) localized in vortex cores of topological superconductors are widely regarded promising building blocks for fault-tolerant quantum computation. However, their unambiguous detection is hindered by the extremely small energy spacing separating them from conventional Caroli-de Gennes-Matricon states. Using a microscopic Bogoliubov-de Gennes approach, we demonstrate that nonmagnetic impurities, rather than suppressing, can substantially enhance the energy gap between MZMs and other vortex core excitations. The robustness of MZMs against local perturbations ensures that while conventional states are shifted by impurity-induced potentials, the MZMs remain intact. This results in a pronounced zero-bias peak in the local density of states. Our results dispute the widespread assumption that large $\Delta /E_{F}$ values—where Δ is the superconducting gap and $E_{F}$ is the Fermi energy—are required to detect MZMs, and instead indicate that purposefully engineered pinning centers in conventional *s*-wave superconductors offer a practical and experimentally accessible alternative.

## Introduction

Majorana zero modes (MZMs), first predicted to exist in vortex cores of a *p*-wave superconductor [1], exhibit several exceptional characteristics that have attracted considerable interest from the broader physics community. MZMs, which can be regarded as “half-fermion” excitations, correspond to 2 degenerate states with energies lying exactly at the midpoint of the superconducting gap. These states are localized, yet exhibit nonlocal behavior: in systems containing a single vortex, the MZM wave functions are distributed between the vortex core and the boundary of the sample [2]. MZMs are topologically protected and separated from nearby states by an energy gap, making them robust against local perturbations and low-energy excitations [3]. They are also predicted to appear at the ends of one-dimensional *p*-wave superconducting wires [4]. Their robustness makes them promising candidates for realizing stable qubits in quantum computation architectures [5–7].

Only a few materials are considered potential *p*-wave superconductors, including Sr_2_RuO_4_ [8], UPt_3_ [9], layered AuSn_4_ [10], and Cu_x_Bi_2_Se_3_ [11]. However, MZMs can also emerge via the proximity effect at interfaces between conventional *s*-wave superconductors and topological insulators [12], or between *s*-wave superconductors and materials exhibiting ferromagnetic Rashba spin-orbit coupling (SOC) [13]. These mechanisms greatly expand the range of materials and hybrid structures in which MZMs can be realized, sparking an intensive search for their experimental detection [2,14,15].

Signatures of MZMs have been reported in various superconducting devices [16–23] and in complicated scanning tunneling microscopy (STM) studies of certain novel materials [24]. However, the experimental observables used to identify MZMs can also arise from nontopological bound states, making unambiguous identification challenging [25,26]. This ambiguity persists even in the recently presented Majorana 1 quantum processor [7]. Zero-bias peaks in the local density of states (LDOS) observed near vortex cores in iron-based superconductors [27–29] have also been attributed to MZMs. However, such peaks may also originate from alternative quasiparticle excitations, such as Caroli-de Gennes-Matricon (CdGM) states [30–33].

One of the major challenges in observing MZMs is the close proximity of neighboring energy levels. In uniform superconductors, the energies of vortex core excitations are given by [1,30]

$$E_{j}=\pm j\Delta^{2}/E_{F},$$ (1)

where Δ is the superconducting gap, $E_{F}$ is the Fermi energy, and *j* is a quantum number–integer valued for *p*-wave pairing and half-integer for *s*-wave pairing. The MZM corresponds to j=0, representing the only doubly degenerate state in the spectrum. It is separated from the nearest excited states by an energy $\Delta E=\Delta^{2}/E_{F}$. Since $\Delta \ll E_{F}$ in most superconductors, this energy is extremely small [34], which poses a major obstacle to the experimental resolution and unambiguous detection of MZMs.

To overcome this problem, researchers have turned to materials with small $E_{F}$ and large Δ [35]. Among these, iron-based superconductors have emerged as leading candidates for MZM observation [27–29,36–48]. However, a serious drawback of iron-based materials is the presence of magnetic impurities from interstitial iron atoms. These impurities suppress the superconducting gap and distort zero-bias peaks, complicating the identification of MZMs [49]. Thus, high-purity single crystals with minimal disorder are essential for reliable conclusions. Furthermore, statistical analysis of bound-state spectra [50] and refined spectroscopy protocols [51] have become crucial tools.

In contrast, nonmagnetic impurities do not affect MZMs due to their topological protection and nonlocal character [1,52]. This distinguishes MZMs from other vortex core states [53,54], offering a possible route for their identification. However, theoretical studies of vortex core states in the presence of pinning centers have shown that while the MZMs remain unperturbed, the energies of other states shift closer together, reducing the visibility of the MZM peak in the LDOS [55]. These results align with the intuitive expectation that disorder leads to spectral crowding and suppression of the visibility of individual states. Nevertheless, experiments on topological superconducting hybrids such as Pb/Co/Si have revealed unexpectedly robust zero-bias peaks, attributed to MZMs, that are separated from other excitations by sizable gaps, presumably arising from magnetic or spin-orbit-induced vortex core textures [56].

This work demonstrates that, contrary to prevailing assumptions and earlier calculations, the mere presence of impurity-induced pinning centers can substantially increase the energy separation between MZMs and other vortex core states, thereby facilitating the identification of the former. With an appropriately chosen impurity potential, in terms of both strength and spatial extent, this energy separation can be substantially enhanced.

**Fig. 1.**
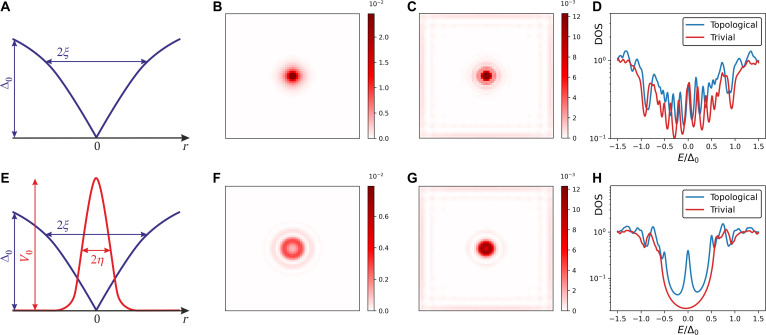
Top panels: Clean system. (A) Illustration of the geometry and parameters of the system. The blue line shows the vortex core solution for the gap function ∣Δ∣, with characteristic width ξ and homogeneous gap magnitude $\Delta_{0}$. (B) Spatial profile of the absolute value of the wave function corresponding to the lowest-energy CdGM state in a nontopological superconductor, $V_{z}=0$. (C) Spatial profile of the absolute value of the wave function corresponding to the MZM in a topological superconductor with $V_{z}=0.5$. (D) Averaged LDOS at the vortex core for topological (blue) and trivial (red) superconductors. Bottom panels: System with pinning potential. (E) Illustration of the geometry and parameters of the system. The red line represents the pinning potential, characterized by its strength $V_{0}$ and width η. The blue line shows the vortex core solution for the gap function ∣Δ∣, with characteristic width ξ and homogeneous gap magnitude $\Delta_{0}$. (F) Spatial profile of the absolute value of the wave function corresponding to the lowest-energy CdGM state in a nontopological superconductor, $V_{z}=0$. (G) Spatial profile of the absolute value of the wave function corresponding to the MZM in a topological superconductor with $V_{z}=0.5$. (H) Averaged LDOS at the vortex core for topological (blue) and trivial (red) superconductors, shown for $V_{0}=0.8$. The impurity width is set equal to the superconducting coherence length, η=ξ=3. The system size is 61×61. In panels (B), (C), (F), and (H), the color bars illustrate the respective distribution density of the wave function for the lower-energy bound state.

Figure 1 illustrates this effect. The top panels (A to D) present results for a clean system: Panel (A) shows the superconducting gap profile within a vortex; panels (B) and (C) display the spatial distribution of the lowest quasiparticle state in trivial and topological superconductors, respectively; and panel (D) depicts the energy dependence of the LDOS. The bottom panels (E to H) show the corresponding results for a system with a pinning potential [indicated by the red line in panel (E)].

These findings suggest that the detection of MZMs becomes considerably more feasible in systems with engineered pinning centers, shifting the focus from materials with high $\Delta /E_{F}$ ratios to more experimentally accessible samples with strategically pinned vortices. Our results are applicable to both intrinsic *p*-wave superconductors and hybrid structures composed of *s*-wave superconductors coupled to materials with strong SOC.

## Results

The distinction between the MZM and the lowest energy CdGM states in a pure superconductor is illustrated in Fig. 1B and C, which show the spatial density of their respective wave functions. The CdGM state is tightly localized around the vortex core (Fig. 1B). In contrast, the MZM exhibits a nonlocal character, being localized both at the vortex core and near the sample boundary (Fig. 1C). It is worth noting that when the system contains 2 or more vortices, the MZM can be distributed or “split” between different vortex cores.

To get a deeper insight, we characterize both the energy and spatial distribution of the quasiparticle states. We calculate the LDOS using

$$\begin{array}{l} N\left(r,\;E\right)=\sum_{n,\sigma }{\left|u_{\sigma }^{\left(n\right)}\left(r\right)\right|}^{2}\delta \left(E-E^{\left(n\right)}\right)\\ \;+{\left|v_{\sigma }^{\left(n\right)}\left(r\right)\right|}^{2}\delta \left(E+E^{\left(n\right)}\right), \end{array}$$ (2)

where the result is smoothed by convoluting with a Lorentzian function of width Γ = 0.01.

Without the impurity, the averaged LDOS $N\left(r\leq \xi ,\;E\right)$ at the vortex core, shown in Fig. 1D of the topological (blue line) and conventional (red line) superconductors, are practically indistinguishable. (In this regard, we note that SOC shifts the energies of the CdGM states, breaking their symmetry around the gap center *E* = 0 [57,58].) However, introducing an impurity substantially modifies the spatial profile of the lowest CdGM state (Fig. 1F) while only slightly perturbing the MZM state (Fig. 1G). This leads to a clear separation of the zero-bias MZM peak at *E* = 0 from the other vortex core states, as clearly evidenced in Fig. 1H.

**Fig. 2.**
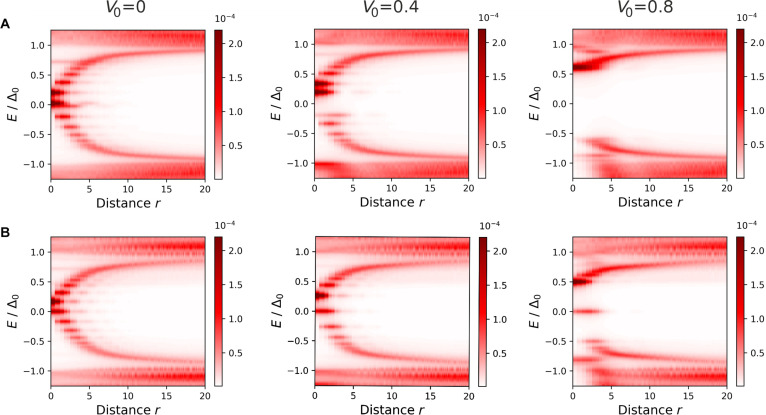
(A) LDOS for vortex core states in a nontopological (trivial) superconductor ($V_{z}=0$), calculated without impurity potential ($V_{0}=0$) and with impurities of strengths $V_{0}=0.4$ and 0.8. (B) LDOS for vortex core states in a topological superconductor ($V_{z}=0.5$), calculated without impurity potential ($V_{0}=0$) and with impurities of strengths $V_{0}=0.4$ and 0.8. The impurity width is set equal to the superconducting coherence length, η=ξ=3. The color bars are adjusted to the maximum value across all the parameter sets considered.

This impurity-induced restructuring of the LDOS $N\left(r,\;E\right)$ is further explored by examining its spatial dependence on the distance from the impurity *r* and shown in Fig. 2. The top and bottom panels correspond to the nontopological (A) and topological (B) superconductors, respectively. The results are presented for different values of the pinning potential: $V_{0}=0$ (no pinning), $V_{0}=0.4$, and $V_{0}=0.8$.

Clearly, the states localized at the vortex core exhibit radial symmetry. The radius of the localization ring for these states increases with energy *E*. In the case of a topological superconductor, the MZM energy is precisely in the middle of the gap, E=0. However, without pinning ($V_{0}=0$), the gap between neighboring excitations is so small that it becomes difficult to distinguish the topological (Fig. 2B) from the nontopological (Fig. 2A) superconductor.

The situation changes when an impurity is introduced into the system. Its potential raises the absolute value of the energies of all the vortex core states, except for the MZM. Consequently, the impurity enhances the energy gap between the MZM and other vortex core states.

**Fig. 3.**
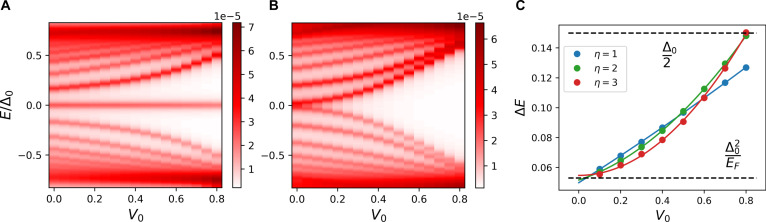
Dependence of the energy levels of the vortex core states on the impurity potential strength $V_{0}$ for the topological (A) and nontopological (B) superconductors. The calculations assume that the impurity width is equal to the superconducting coherence length, η=ξ=3. The color bars indicate the value of DOS. (C) The variation of the energy gap ΔE between the MZM and other vortex core states as a function of pinning strength $V_{0}$, computed for impurity widths η=1, 2, and 3. The lines between points are shown as guides to the eye.

The dependence of the energy levels of all vortex states on the impurity potential strength $V_{0}$ is shown in Fig. 3. Except for the MZM, the energies of all states in both the topological (Fig. 3A) and nontopological (Fig. 3B) superconductors shift away from the center of the superconducting gap at *E* = 0. Figure 3C illustrates how the energy gap ΔE, which separates the MZM from the other vortex states, varies with $V_{0}$. Although this dependence changes with the width η of the impurity potential, it remains monotonic.

**Fig. 4.**
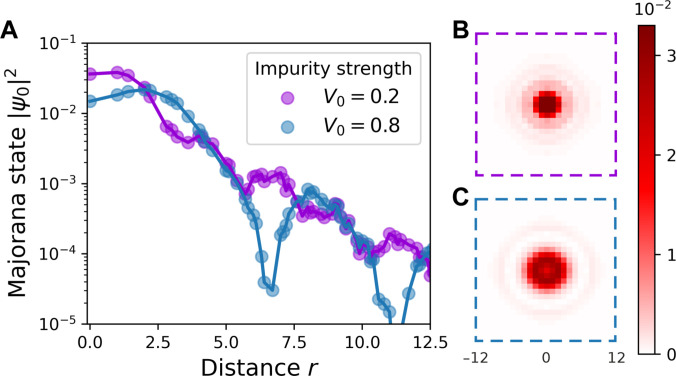
(A) Radial dependence of the MZM wave function as a function of distance *r* from the vortex core, calculated for impurity strengths $V_{0}=0.2$ and 0.8. Panels (B) and (C) show density plots of the spatial distribution of the MZM wave function for $V_{0}=0.2$ and 0.8, respectively. The impurity width is set equal to the superconducting coherence length, η=ξ=3. The color bar is normalized to the maximum value of the density of the wave function for both panels.

We note in passing that the spatial profile of the MZM wave functions differs qualitatively from that of the lowest energy CdGM state—the radial dependence of the MZM wave function exhibits pronounced oscillations (Fig. 4). These oscillations are also visible as scars in the density plot shown in Fig. 1C. Both the period and the amplitude of these oscillations are notably dependent on the impurity strength $V_{0}$.

We emphasize that our calculations predict an increase in the energy spacing ΔE for systems with electron-type charge carriers, which occurs when the chemical potential is negative, μ<0 (μ=0 corresponds to the center of the conduction band). In contrast, for systems with hole-type carriers (μ>0), the impurity potential has the opposite effect: it shifts the energies of the vortex core states toward the center of the superconducting gap, E=0. This reversed behavior was previously reported in Ref. [55], where it was concluded that impurities may further suppress the visibility of MZMs. A similar effect is observed when the impurity potential becomes attractive for electrons ($V_{0}<0$), again leading to a shift of the vortex core states toward E=0.

At first glance, these findings suggest that the outcome strongly depends on the impurity type and the nature of the charge carriers. However, this interpretation is misleading. Calculations predicting the reverse effect also rely on the same approximation (Eq. 9), which becomes invalid when the impurity attracts quasiparticles ($V_{0}<0$) or the system becomes hole-like (μ>0). In such cases, the vortex is repelled by the impurity, rendering the solution based on Eq. 9 unstable. Therefore, adding “usual” impurities $V_{0}>0$ can only enhance the visibility of MZMs, not suppress it, as in this case the vortex is strongly pinned to the impurity, which effectively acts as a local potential barrier that pushes the higher-energy CdGM states away from MZM.

## Conclusion

In this work, we have investigated the effect of an impurity potential on MZMs localized in the vortex core of a hybrid topological superconductor, composed of a conventional *s*-wave superconductor, a Rashba spin-orbit coupled semiconductor, and a ferromagnetic material.

Our direct numerical calculations based on a microscopic model show that introducing nonmagnetic impurities increases the energy separation between MZMs and other vortex core states, while the MZMs themselves remain robust. This finding substantially relaxes the conventional requirement for a high $\Delta /E_{F}$ ratio, which has traditionally hindered the experimental detection of MZMs due to the typically small energy spacing between MZMs and low-energy in-gap excitations. Our results suggest that, rather than relying on materials with large $\Delta /E_{F}$ ratios (such as iron-based superconductors), it is feasible to observe MZMs in hybrid topological systems based on conventional *s*-wave superconductors containing single, strategically engineered nonmagnetic impurities.

Results of the work remain valid as long as the width of the impurity lies within the range $1/k_{F}≲\eta ≲\xi$, ensuring that it does not exceed the size of the vortex core and $V_{0}$ is high enough to suppress superconducting gap.

Our 2-dimensional model captures the essential surface physics governing topological superconductivity and MZM formation in relevant 3-dimensional (3D) platforms. It is directly applicable to 3D samples with columnar defects, where MZMs remain localized at the vortex ends. Surface defects, such as those created by focused nanoscale ion beams or arising from band bending [49,59,60], are likewise expected to yield a comparable widening of the minigap in the surface LDOS, owing to the suppressed contribution from relevant bulk states near the surface.

We finally note that strongly pinned vortices are not ideal for direct motion-based braiding schemes proposed for quantum computations; nevertheless, MZM states at such vortices can still be controlled via alternative approaches. For instance, manipulation can be achieved by controlling the MZM’s entangled partner at the sample boundary. This strategy enables control of MZM’s via local gating or phase biasing without moving the vortices themselves [61]. In this context, the proposed mechanism for reliable MZM detection in readily available materials could be essential for designing MZM-based quantum computation schemes.

## Methods

### The model of topological superconductor

We consider a hybrid topological superconductor that comprises layers of a superconductor with *s*-wave pairing symmetry, a semiconductor with Rashba SOC, and a ferromagnetic insulator. Such a system is described using the discrete model Hamiltonian [62]

$$H=H_{s}+H_{\mathrm{SOC}}+H_{m},$$ (3)

where the Hamiltonian of the superconducting subsystem is written in the mean-field approximation as

$$H_{s}=\sum_{\langle i,\;j\rangle ,\sigma }t_{\mathrm{ij}}c_{\mathrm{i\sigma}}^{†}c_{\mathrm{j\sigma}}+\sum_{i}\left(\Delta_{i}c_{i↑}^{†}c_{i↓}^{†}+h.c.\right),$$ (4)

with $c_{i\sigma }$ being the electron operators corresponding to lattice site *i* and spin σ. The hopping elements $t_{\mathrm{ij}}=t$ are nonzero only for nearest neighbors, and $\Delta_{i}$ is the gap function at site *i*. The magnetic part is described by the Hamiltonian

$$H_{m}=V_{z}\sum_{i}\left(c_{i↑}^{†}c_{i↑}-c_{i↓}^{†}c_{i↓}\right),$$ (5)

with $V_{z}$ characterizing the Zeeman splitting. Finally, the Rashba interaction Hamiltonian is given by

$$\begin{array}{l} H_{\mathrm{SOC}}=\frac{\alpha }{2}\sum_{i}\left[\left(c_{i-e_{x}↓}^{†}c_{i↑}-c_{i+e_{x}↓}^{†}c_{i↑}\right)\right.+\\ \;i\left.\left(c_{i-e_{y}↓}^{†}c_{i↑}-c_{i+e_{y}↓}^{†}c_{i↑}\right)+h.c.\right], \end{array}$$ (6)

where α is the SOC hopping amplitude responsible for spin-flip hopping between nearest neighbors.

We also consider an additional contribution due to an impurity located at position $r_{0}$, described by a Gaussian potential

$$V_{i}=V_{0}\;\exp\left(-\frac{{\left|r_{i}-r_{0}\right|}^{2}}{2\eta^{2}}\right),$$ (7)

where $V_{0}$ denotes the strength of the impurity and η its spatial extent. Compared to the Fu–Kane model, the Hamiltonian (Eq. 3) includes extra kinetic and Zeeman terms, allowing a trivial phase.

The vortex core states are studied by solving the Bogoliubov-de Gennes (BdG) equations corresponding to the model Hamiltonian (Eq. 3). The approach follows the methodology used for the analysis of CdGM states in conventional *s*-wave superconductors [61–65]. In block-matrix form, the BdG equations are written as

$$\left[\begin{array}{cccc} {\hat{h}}_{+} & \hat{g} & 0 & \hat{\Delta }\\ -\hat{g} & {\hat{h}}_{-} & \hat{\Delta } & 0\\ 0 & {\hat{\Delta }}^{*} & -{\hat{h}}_{+}^{*} & -{\hat{g}}^{*}\\ {\hat{\Delta }}^{*} & 0 & {\hat{g}}^{*} & -{\hat{h}}_{-}^{*} \end{array}\right]\left(\begin{array}{c} {\vec{u}}_{+}\\ {\vec{u}}_{-}\\ {\vec{v}}_{+}\\ {\vec{v}}_{-} \end{array}\right)=E\left(\begin{array}{c} {\vec{u}}_{+}\\ {\vec{u}}_{-}\\ {\vec{v}}_{+}\\ {\vec{v}}_{-} \end{array}\right),$$ (8)

where the components of the eigenvectors ${\vec{u}}_{\sigma }$ and ${\vec{v}}_{\sigma }$ correspond to lattice sites *i*. The elements of the block matrices are defined as ${\left[\hat{h}\pm \right]}_{\mathrm{ij}}=t_{\mathrm{ij}}+\left(V_{i}\pm V_{z}-\mu \right)\delta_{\mathrm{ij}}$, with $\delta_{\mathrm{ij}}$ being the Kronecker delta; ${\left[\hat{g}\right]}_{\mathrm{ij}}=\alpha_{\mathrm{ij}}$, where $\alpha_{\mathrm{ij}}$ takes values of ±α/2 for right/left neighbors and ±iα/2 for upper/lower neighbors; and ${\left[\Delta \right]}_{\mathrm{ij}}=\Delta_{i}\delta_{\mathrm{ij}}$.

### Details of calculations

To obtain the vortex solution, Eq. 8 must be solved in the presence of a magnetic field, which is incorporated by introducing the Peierls phase factor into the hopping integrals as $t_{\mathrm{ij}}\exp\left(i\int_{r_{i}}^{r_{j}}A\left(r\right)dr/\phi_{0}\right)$, where $A\left(r\right)$ is the vector potential of the magnetic field and $\phi_{0}$ is the flux quantum. The solution for the vortex must satisfy the self-consistency conditions for $\Delta_{i}$ as well as for the field A. Achieving full self-consistency in the presence of the magnetic field requires 2 iterative cycles [63].

Solving the BdG equations exactly is computationally demanding. To reduce the computational load, we employ a simplified approach in which the vortex solution is approximated as follows:

$$\Delta_{i}=\Delta_{0}e^{i\theta_{i}}\tanh\left(\frac{|r_{i}-r_{0}|}{\xi }\right),$$ (9)

where $\Delta_{0}$ represents the homogeneous gap magnitude, $|r_{i}-r_{0}|$ is the distance from the vortex center, ξ denotes the coherence length, and $\theta_{i}$ is the polar angle at the lattice site *i*. Comparison with the exact self-consistent solution shows that this approximation is highly accurate when the superconductor is well within the type II regime [63,66]. Another necessary condition is that the impurity potential is weak, satisfying $V_{0}\ll W$, where W=8t is the bandwidth, and that η≲ξ. However, we employ the exact self-consistent solution to confirm whether the vortex is attracted to the impurity, acting as a pinning center, or repelled by it. We also note that a solution of the BdG equations depends on the system temperature (*T* = 0 in our calculations), which enters Eq. 9 via temperature-dependent quantities $\Delta_{0}$ and ξ.

Numerical calculations are performed on a finite sample of size 61 × 61 (measured in units of the lattice constant *a*), with boundary conditions set as u=v=0. All energy values are expressed in units of the hopping constant, with *t* = 1 serving as the energy scale. We set the SOC constant to α=1.2 in accordance with Ref. [61], which demonstrated the existence of the topological phase at this level of SOC, and the chemical potential to μ=−3.5, which corresponds to a shallow electron-like band.

We also take $\Delta_{0}=0.3$ and ξ=3, which is substantially smaller than the sample size *L*. The relative values of the chosen parameters are illustrated in Fig. 1E, which plots the profile of the gap function in the vortex core (blue line) and the impurity potential (red line). The choice ensures the applicability of the approximate solution (Eq. 9).

The Zeeman splitting $V_{z}$ governs the topological nature of the vortex core states. When $V_{z}=0$, the system is a trivial superconductor featuring CdGM states localized around the vortex core. However, when Zeeman splitting satisfies the condition ${\left(4t-|\mu |\right)}^{2}+\Delta_{0}^{2}<V_{z}^{2}<\mu^{2}+\Delta_{0}^{2}$, the system becomes a topological superconductor that hosts MZMs near the vortex core [67]. In the present study, we choose $V_{z}=0.5$, which falls within this range, thus placing the system in the topological superconductor regime. The calculations are done using the high performance computing facilities [68].

## Data Availability

All data needed to evaluate the conclusions in the paper are freely available at https://gitlab.com/center-quantum-metamaterials/impurity_and_mzm/.
